# The effect of community-based programs on diabetes prevention in low- and middle-income countries: a systematic review and meta-analysis

**DOI:** 10.1186/s12992-019-0451-4

**Published:** 2019-02-01

**Authors:** Maryam Shirinzadeh, Babak Afshin-Pour, Ricardo Angeles, Jessica Gaber, Gina Agarwal

**Affiliations:** 1 Department of Health Research Methodology, McMaster University, 1280 Main St W, Hamilton, Ontario L8S 4L8 Canada; 2 Biosymetric Inc, 180 John St, Toronto, Ontario M5T 1X5 Canada; 3 Department of Family Medicine, McMaster University, 1280 Main St W, Hamilton, Ontario L8S 4L8 Canada; 4 Department of Family Medicine, and Department of Health Research Methods, Evidence and Impact, McMaster University, 1280 Main St W, Hamilton, Ontario L8S 4L8 Canada

**Keywords:** Diabetes, Community-based program, Incidence rate, HbA1C, Systematic review, Meta-analysis, Low and middle income countries

## Abstract

**Background:**

The increasing prevalence of type 2 diabetes mellitus (T2DM) can have a substantial impact in low- and middle-income countries (LMICs). Community-based programs addressing diet, physical activity, and health behaviors have shown significant benefits on the prevention and management of T2DM, mainly in high-income countries. However, their effects on preventing T2DM in the at-risk population of LMICs have not been thoroughly evaluated.

**Methods:**

The Cochrane Library (CENTRAL), MEDLINE, EMBASE and two clinical trial registries were searched to identify eligible studies. We applied a 10 years limit (from 01 Jan 2008 to 06 Mar 2018) on English language literature. We included randomized controlled trials (RCTs) with programs focused on lifestyle changes such as weight loss and/or physical activity increase, without pharmacological treatments, which aimed to alter incidence of diabetes or one of the T2DM risk factors, of at least 6 months duration based on follow-up, conducted in LMICs.

**Results:**

Six RCTs randomizing 2574 people were included. The risk of developing diabetes in the intervention groups reduced more than 40%, RR (0.57 [0.30, 1.06]), for 1921 participants (moderate quality evidence), though it was not statistically significant. Significant differences were observed in weight, body mass index, and waist circumference change in favor of community-based programs from baseline, (MD [95% CI]; − 2.30 [− 3.40, − 1.19], *p* < 0.01, I2 = 87%), (MD [95% CI]; − 1.27 [− 2.10, − 0.44], *p* < 0.01, I2 = 96%), and (MD [95% CI]; − 1.66 [− 3.17, − 0.15], *p* = 0.03, I2 = 95%), respectively. The pooled effect showed a significant reduction in fasting blood glucose and HbA1C measurements in favor of the intervention (MD [95% CI]; − 4.94 [− 8.33, − 1.55], *p* < 0.01, I2 = 62%), (MD [95% CI]; − 1.17 [− 1.51, − 0.82], *p* < 0.01, I2 = 46%), respectively. No significant difference was observed in 2-h blood glucose values, systolic or diastolic blood pressure change between the two groups.

**Conclusion:**

Based on available literature, evidence suggests that community-based interventions may reduce the incidence rate of T2DM and may positively affect anthropometric indices and HbA1C. Due to the heterogeneity observed between trials we recommend more well-designed RCTs with longer follow-up durations be executed, to confirm whether community-based interventions lead to reduced T2DM events in the at-risk population of LMIC settings.

**Electronic supplementary material:**

The online version of this article (10.1186/s12992-019-0451-4) contains supplementary material, which is available to authorized users.

## Background

Diabetes is a common chronic disease worldwide; in 2011, it affected 366 million people [1, 2]. It is estimated that 592 million people will have the condition by 2035 [3]. This growing prevalence is related to increasing economic growth, urbanization, and lifestyle alteration characterized by risk factors such as obesity and sedentary activity [4, 5]. Prediabetes, i.e. impaired fasting blood glucose or impaired glucose tolerance, often occurs about 5 years before the development of type 2 diabetes mellitus (T2DM) [6]. People with prediabetes are at 5 to 15% greater risk of progression to T2DM [7].

Large randomized controlled trials (RCTs) from Finland, India, China, and the US have shown that lifestyle interventions can decrease the incidence of T2DM from 58 to 29% in high-risk populations [8–11], with maintenance up to 20 years [12, 13]. Currently, 7 of the top 10 countries with the greatest number of people living with diabetes are low- and middle-income countries (LMICs), including China, India, Brazil, Pakistan, Indonesia, and Bangladesh [3, 14–17]. In the last decade, the prevalence of diabetes has escalated more in LMICs compared to high-income countries (HICs). The numbers of people with diabetes differ substantially by country income group [3]. By 2030, the number of people with diabetes is projected to increase by 92% in low-income countries, 57% in lower-middle income countries, 46% in upper-middle income countries, and 25% in HICs. The rate of increase is inversely associated with the current income status of countries [3].

One of the challenges in many countries is to establish effective and low-cost interventions to prevent the development of T2DM that can be successfully implemented and sustained [18]. Community-based programs are practical, relatively low-resource, and often involve educational programs aiming at lifestyle change. They address various aspects of health, including diet, physical activity, and health behaviors and have demonstrated significant benefits for the management of chronic diseases such as cardiovascular disease (CVD) and T2DM [19]. Community-based programs are appealing, since they can reach people outside of conventional healthcare settings, and usually target all groups in the community. If a program succeeds with a positive effect on behavioral change, it probably can achieve considerable and widespread risk reduction in the community [20]. In recent years, several systematic reviews have reported on the positive effects of diabetes prevention programs, mainly in HICs [20–22], but are now drawing more attention in LMICs. The data encourage policymakers at local and national levels to collaborate in order to mitigate the rising prevalence of T2DM in their populations. Community-based programs mainly provide health behavioral interventions through group-based (and sometimes also individual) educational sessions to achieve health-related behavioral modification in the communities. Since busy healthcare settings do not have adequate capacity to offer intensive behavioral interventions [23], programs with greater expertise and resources to offer effective interventions and enhance health-related behavioral modification should be offered to the whole community [24].

Three landmark clinical trials of people at risk of diabetes have indicated a remarkable relative risk reduction in the progression to T2DM with health-related behavioral modification [11–13, 25–27]. However, it is still unclear how these behavioral changes influence the risk of complications related to T2DM. Despite the continued development and use of community-based programs, there is currently limited evidence to support or refute their effectiveness in LMICs. Most studies reported in recent systematic reviews have been based in HICs, while few studies based in LMICs have been performed [21, 22, 28]. One review did cover diabetes prevention programs in developing countries [29], but the researchers performed literature searches only until September 2009. To our knowledge, few reviewers to date have tried to assess the effectiveness of community-based programs in risk reduction of T2DM in LMICs. Generalizing evidence from HICs to LMICs needs to be considered with caution given cultural, ethnic, and economic differences, as well as the differences in targeted populations [30]. This systematic review and meta-analysis aims to review the last decade of evidence on the effectiveness of community-based programs to prevent or reduce the risk of developing T2DM in at-risk populations in LMICs.

## Methods

### Types of studies and participants

We included all randomized controlled trials (RCTs) of at least 6 months duration published from 01 January 2008 to 06 March 2018 reporting on the evaluation of community-based programs/interventions for the prevention or risk reduction of T2DM compared with no program or standard treatment, aimed at non-diabetic adult populations (≥ 18 years of age) at risk of type 2 diabetes who live in LMICs as defined by the World Bank country classification at the time of the data collection [31]. Methods of T2DM risk assessment included the fasting glucose test, glucose tolerance test, HbA1C, and Diabetes Risk Score based on recognized assessment tools (American Diabetes Association score, Finnish Diabetes Risk Score, Australian Diabetes Risk Score, Indian Diabetes Risk Score, or Canadian Diabetes Risk Score). We excluded trials where there was evidence that researchers included individuals with diabetes, CVD, any other major health conditions, current pregnancy, or breastfeeding.

### Types of interventions and outcomes

Included interventions were community-based programs without pharmacological treatments which included behavioral modification like dietary advice, weight loss, or increasing activity level, and aimed to alter the incidence of diabetes or a diabetes risk factor such as weight, blood pressure, or glycemic control. Community-based programs were defined as those targeting whole populations or specific groups (for example an age group, e.g., older adults) living within a specific geographic area and which reached populations outside healthcare/clinical settings. These settings may have included workplaces, churches, schools, etc. The size of target areas was not specified. Comparison was defined as no intervention or standard treatment for the control group. The primary outcome of interest was the incidence of T2DM. Secondary outcomes included change in anthropometric indices (weight, body mass index [BMI], waist circumference), glycemic control change (fasting blood glucose, 2-h blood glucose, HbA1C), and blood pressure change.

### Search strategy, identification and selection of studies

We identified studies through systematic searches of the Cochrane Library, MEDLINE, and EMBASE for the period of 01 January 2008 to 06 March 2018. We included only studies published in the English language. We also searched clinical trial registries (Clinicaltrials.gov and WHO International Clinical Trials Registry) for trials that had outcomes but had yet to be published. Furthermore, we checked the reference lists of all primary eligible studies and review articles for additional references. We excluded trials: where the intervention or control group included the administration of any pharmacological agent, that applied diet advice through single-food or dietary supplements (e.g., vitamin D supplement), and that had identical interventions applied with different approaches (e.g., the same intervention applied in more-intensive or less-intensive ways). We also excluded trials where none of our primary or secondary outcome measures were reported in the publication and contact with the corresponding author was not provided in the supplementary data. The search strategy is detailed in Additional file 1.

**Fig. 1:**
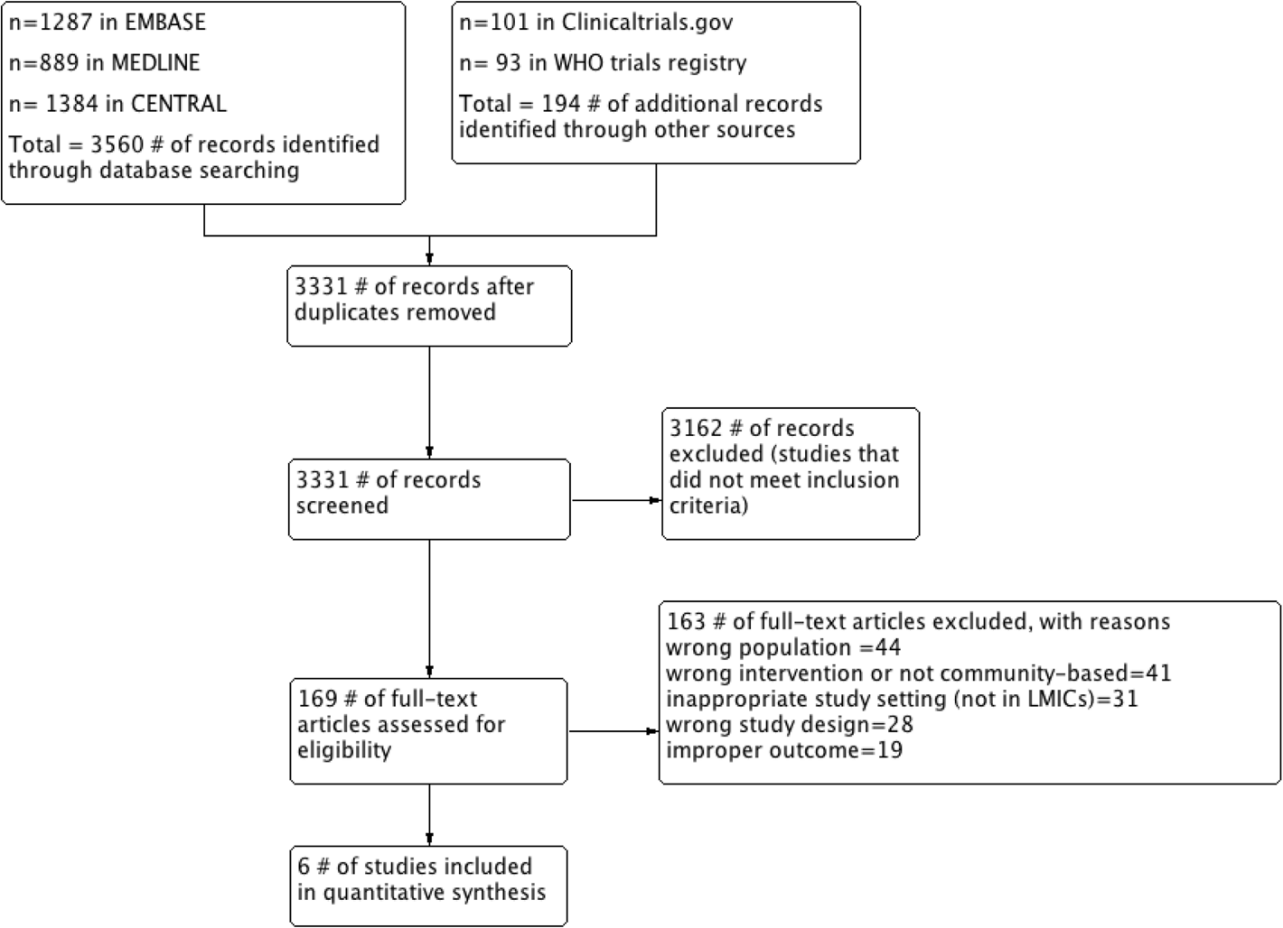
Study flow diagram

Citations and abstracts of all retrieved studies were imported into EndNote X8 citation management software. Duplicates were removed, and the remaining studies were assessed for eligibility criteria by the Rayyan QCRI web application. Two reviewers independently scanned titles and abstracts of all publications identified from searches and excluded studies that were obviously irrelevant and did not meet the inclusion criteria. Once full-text copies of all potentially relevant studies had been retrieved, the same two reviewers independently assessed potentially eligible trials for inclusion and resolved any disagreements through discussion. We contacted trial authors for additional information if necessary. The study selection process was documented using a PRISMA study selection flow chart (Fig. 1); List of the excluded studies with reasons for exclusion are detailed in the characteristics of excluded studies table (see Additional file 2).

### Data collection, data synthesis and analysis

For data extraction, three reviewers achieved consensus on which data to extract from included studies and the data extraction form have been prepared. We used Microsoft Excel to track study characteristics and outcome data. Two reviewers independently extracted study characteristics from the included studies and filled the data extraction form in excel. All disagreements have been resolved by discussion. For data entry in the software, first one reviewer transferred data into the Review Manager 5.3 software (RevMan 5.3) [32]. A second reviewer double-checked that whether data had been entered accurately by comparing it with study characteristics, and data extraction form. We also used Review Manager 5.3 to conduct the analyses. We reported dichotomous outcomes as risk ratios (RRs) with 95% confidence intervals (CIs). For continuous outcomes, we calculated mean differences (MDs) with 95% CIs when the studies used the same scale. When studies used different measurement units to assess the same outcome (e.g., blood glucose), we calculated and pooled effect sizes using the standardized mean differences (SMDs) with 95% CI [33]. Where standard deviations (SDs) were missing, but CIs or *P* values were available, we obtained SDs by calculation using RevMan 5.3. We contacted study authors to obtain missing outcome data.

Heterogeneity was assessed by visual inspection of a forest plot along with consideration of the chi-squared test and the I^2^ statistic. We interpreted the I^2^ estimate as “might not be important” (0 to 40%), “moderate” (30 to 60%), “substantial” (50 to 90%) or “considerable” (75 to 100%) as recommended in the Cochrane Handbook for Systematic Reviews of Interventions [33]. We planned to carry out subgroup analyses of studies that used individual randomization versus those using cluster randomization to explore heterogeneity. We planned to use funnel plots to evaluate potential small-study biases and publication bias if we included 10 or more trials investigating a particular outcome. Due to the limited number of included RCTs (6 studies), we were not able to construct funnel plots in order to assess reporting bias. We performed data synthesis according to recommendations in the Cochrane Handbook for Systematic Reviews of Interventions [33], and statistical analyses using RevMan 5.3. We used both random-effects and fixed-effect models for meta-analyses [32]. We presented results from the random-effects model when heterogeneity was high and from fixed-effects when heterogeneity was low [32]. We conducted sensitivity analyses to examine the effect of removing studies at high risk of bias from the analyses on the pooled results in the domain of allocation concealment.

### Assessment of risk of bias and the quality of the evidence

**Fig. 2:**
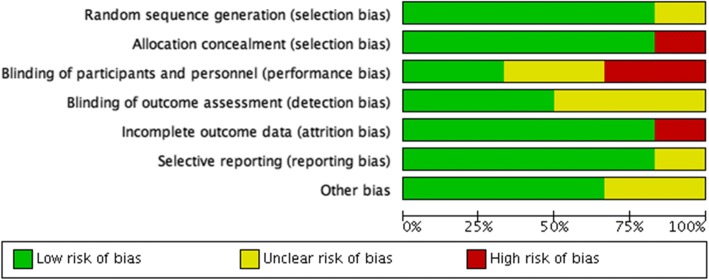
Risk of bias graph; authors’ judgements about each risk of bias item presented as percentages across all included studies

**Fig. 3:**
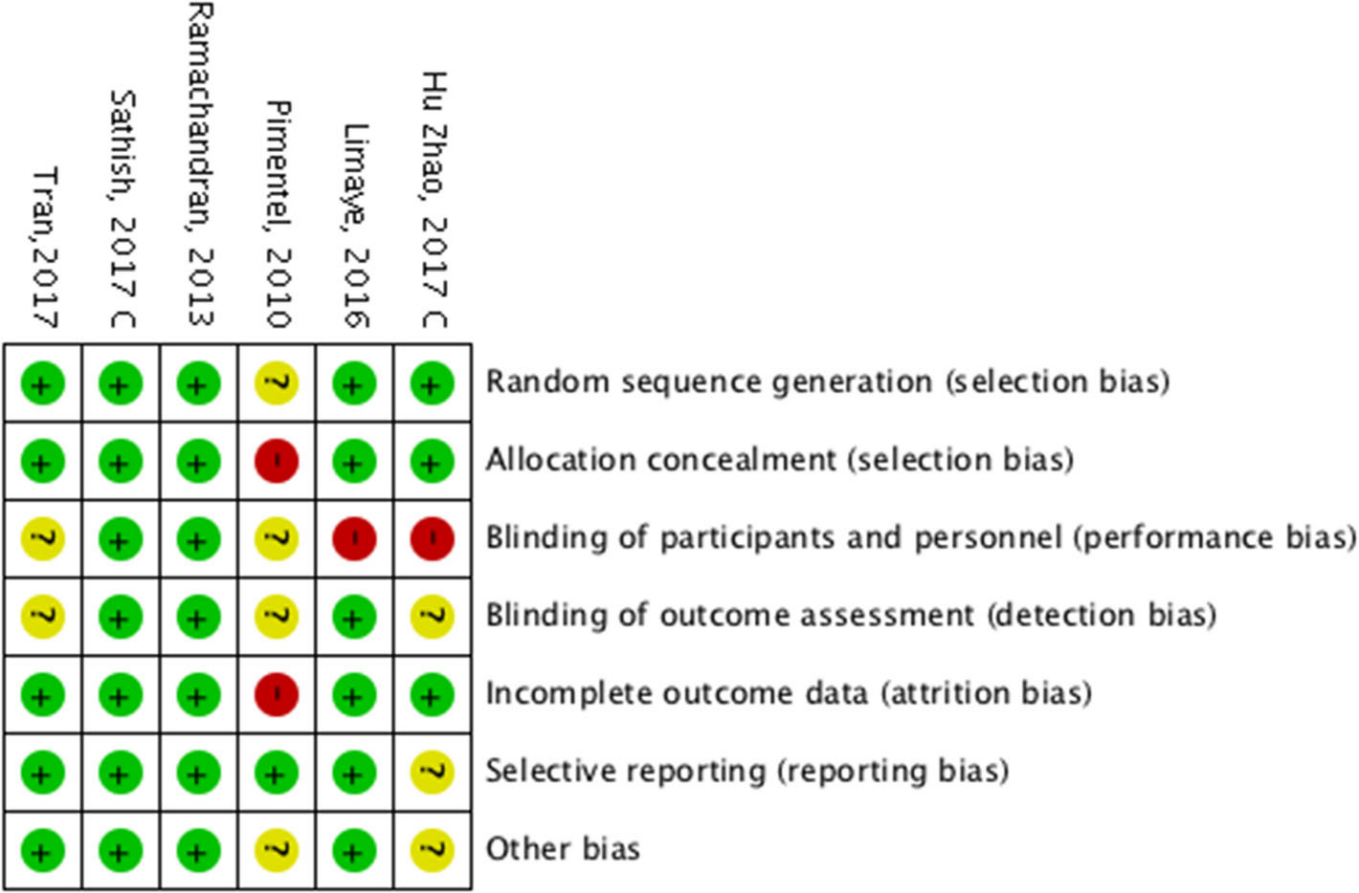
Risk of bias summary; authors’ judgements about each risk of bias item for each included study

Two reviewers assessed the risk of bias of each included trial independently. We resolved any disagreements by discussion. We used the Cochrane Handbook for Systematic Reviews of Interventions [33] to assess the risk of bias according to the following seven domains: random sequence generation, allocation concealment, blinding of outcome assessment, incomplete outcome data, selective outcome reporting, and other bias (for cluster-randomized trials, we assessed the cluster-specific risks of bias) [33]. We graded each risk of bias criteria as either low, high, or unclear risk of bias [33] and summarized our judgements across different studies for each domain listed in Fig. 2 and Fig. 3.

We evaluated the quality of the evidence using the GRADE approach [34], and GRADEpro GDT software [35]. The result is presented in Fig. 5. The level of quality is judged on a four-point scale: high, moderate, low, and very low [34]. We downgraded the evidence from studies by one or two levels after assessing the following domains: risk of bias in the studies, indirectness of the evidence, inconsistency in the results, imprecision of the results, possibility of publication bias, and consideration of upgrading possibility [34].

## Results

### Description of studies

#### Results of the search, excluded and included studies

The database and other resource searches yielded 3331 titles of potentially relevant records after duplicates were removed. Of these, 6 RCTs met the inclusion criteria. The study selection process is summarized in the PRISMA flow diagram shown in Fig. 1. Table 1 gives more information about the characteristics of included studies. Further details and reasons for exclusion of the excluded studies [36–65] are in Additional file 2.

**Table 1:** Characteristics of included studies

First author, year published	Country, Study location	No. of participants	Age (y), mean(SD)	Interventions	Methods & Follow-up duration	Outcomes
Sathish, 2017	India, Kerala state	1007	47 (7.5)	Intervention arm received; -Eleven peer led small group sessions -Two diabetes prevention education sessions -Participant handbook and workbook -Health education booklet Control arm received only health education booklet	Cluster randomized controlled trial Maximum follow-up: 24 months	Incidence of diabetes
Tran, 2017	Vietnam, Hanam province, 10 communes	417	57 (5)	Intervention group received: four components 1-four educational session 2- an information booklet 3- a resistance band 4- a walking group with a leader. Control group was on the waiting list to receive the intervention after completion of the post-test data collection.	Cluster randomized controlled trial Maximum follow-up: 6 months	Anthropometric indices, glycemic control, Blood pressure
Hu Zhao, 2017	China, Hunan Province, 42 villages of Yiyang City	434	69.3 (6.5)	Intervention Group were given an intense synthetic intervention: The synthetic intervention model included lifestyle education, lifestyle intervention, training for the self-monitoring of blood glucose and setting up a Help Each Other Group (HEOG). Control group were given standard primary care.	Cluster randomized controlled trial Maximum follow-up: 12 months	Incidence of diabetes, blood pressure, anthropometric indices, glycemic control
Limaye, 2016	India, Pune, two multinational IT industries	265	36 (9)	Intervention group received LIMIT program (LIfestyle Modification in IT); mobile phone and e-mail (virtual assistance)-based lifestyle intervention using combination of messages and emails to promote healthy lifestyle behaviours. Control group received no educational program.	Individuals randomized controlled trial Maximum follow-up: 12 months	Blood pressure, anthropometric indices, glycemic control
Ramachandran, 2013	India, 10 sites in southeast India	537	46 (4.7)	Intervention group: individually tailored mobile phone messaging including personalized education and motivation about healthy lifestyle principles, diet and physical activity. Control group: standard lifestyle modification advice at baseline	Individuals randomized controlled trial Maximum follow-up: 24 months	Incidence of diabetes, blood pressure, anthropometric indices
Pimentel, 2010	Brazil, Lins city in southeast Brazil	67	56 (12)	Intervention group received the dietary intervention consisted of discussion-format group sessions twice per month and individual sessions once per month to improve healthy behaviours. Control group received no program	Individuals randomized controlled trial Maximum follow-up: 12 months	Anthropometric indices, glycemic control

We included six randomized controlled trials published between 2010 and 2017: three trials conducted in India [66–68], one in China [69], one in Brazil [70], and one in Vietnam [71]. Details of the methods, participants, intervention, comparison group, and outcome measures for each included trial are provided in Table 1. The randomization unit for four trials was the individual participants; two trials [68, 69] used the cluster randomization method. Of these two cluster RCTs, one study [68] pointed out that the result had been adjusted for clustering effect but did not mention the intraclass correlation coefficient (ICC) value, while the other [69] adjusted the results for age, sex, and other characteristics, but did not report applying adjustment aimed at the clustering effect. All trials recruited participants during the implementation of a community-based program. All interventions delivered dietary advice, health behavior guidance, or recommendations for physical activity. The educational approaches varied across the trials from educational sessions to text messages. Duration of follow-up in most of the studies was 12 months while one followed up at 6 months [71] and one at 24 months [67].

#### Risk of bias across the studies

Two studies [67, 68] were free from risk of bias in all domains. Three trials introduced an unclear risk of biases in some domains [66, 69, 71] and only one study [70] was judged to be high risk in two domains (Fig. 2 and Fig. 3).

*Allocation:* The generation of allocation sequence was sufficiently described in five trials [66–69, 71]. The random sequence generation was generated by a random numbers table or a computer-generated sequence. Consequently, we considered them low risk of bias. One trial [70] did not describe its method of randomization, and there were some significant differences in baseline characteristics between two groups like age and fasting blood sugar (FBS), which introduced a high possibility of risk of bias in allocation.

*Blinding:* Since double-blinding was not easily attainable in the included trials due to the type of interventions, only two studies reported blinding of personnel and participants. However, outcome assessment could be blinded to participant allocation, but only three trials indicated that they took measures to do so [66, 67]. We considered trials low risk if knowledge of the intervention to which participants were allocated was unlikely to introduce bias, or there was no significant difference between two arms in the reported outcomes. Regarding some of our outcomes of interest, the incidence rate of diabetes and biochemical measurements were unlikely to be influenced by lack of blinding. Therefore, we considered all studies low risk of bias for these outcomes when assessing the quality of evidence. However, anthropometric measurements may have been affected by performance bias in case of lack of blinding. (Fig. 2 and Fig. 3).

*Incomplete outcome data:* We considered a trial low risk when there were no missing data; reasons for missing outcome data were likely to be irrelevant to the target results, or missing data were reasonably well balanced between groups. Five trials appeared to be free of risk of bias [66–69, 71]. One trial was judged to be high risk due to imbalanced missing data between two groups [70].

*Selective reporting:* We considered trials low risk if findings were clearly reported and the report included adequate data which could be entered for meta-analysis of the reported outcomes. Five trials were judged to be low risk in this domain [66–68, 70, 71] and one trial [69] was considered unclear in risk of bias, since the results did not clearly state in one outcome.

*Other potential sources of biases:* We considered trials low risk if the trials appeared to be free of other sources of bias. We assessed cluster-specific biases in two included cluster randomized trials [68, 69]. We specified them by “C” in their ID (“Hu Zhao, 2017 C” and “Sathish 2017, C”) in meta-analysis tables and figures. Sathish T, [68] adjusted outcomes for the effect of clustering without reporting the ICC. Hu et al., [69] did not state if they adjusted for clustering or the ICC value, but the study mentioned that its sample size (214 in intervention arm and 220 in control arm at the beginning of the study) was about 35% more than what was needed for individual randomization (158 for each group). Thus, we considered it unclear of risk of bias in this domain.

### Effects of the intervention and assessment of the evidence

#### Primary outcome

##### Incidence of diabetes

**Fig. 4:**
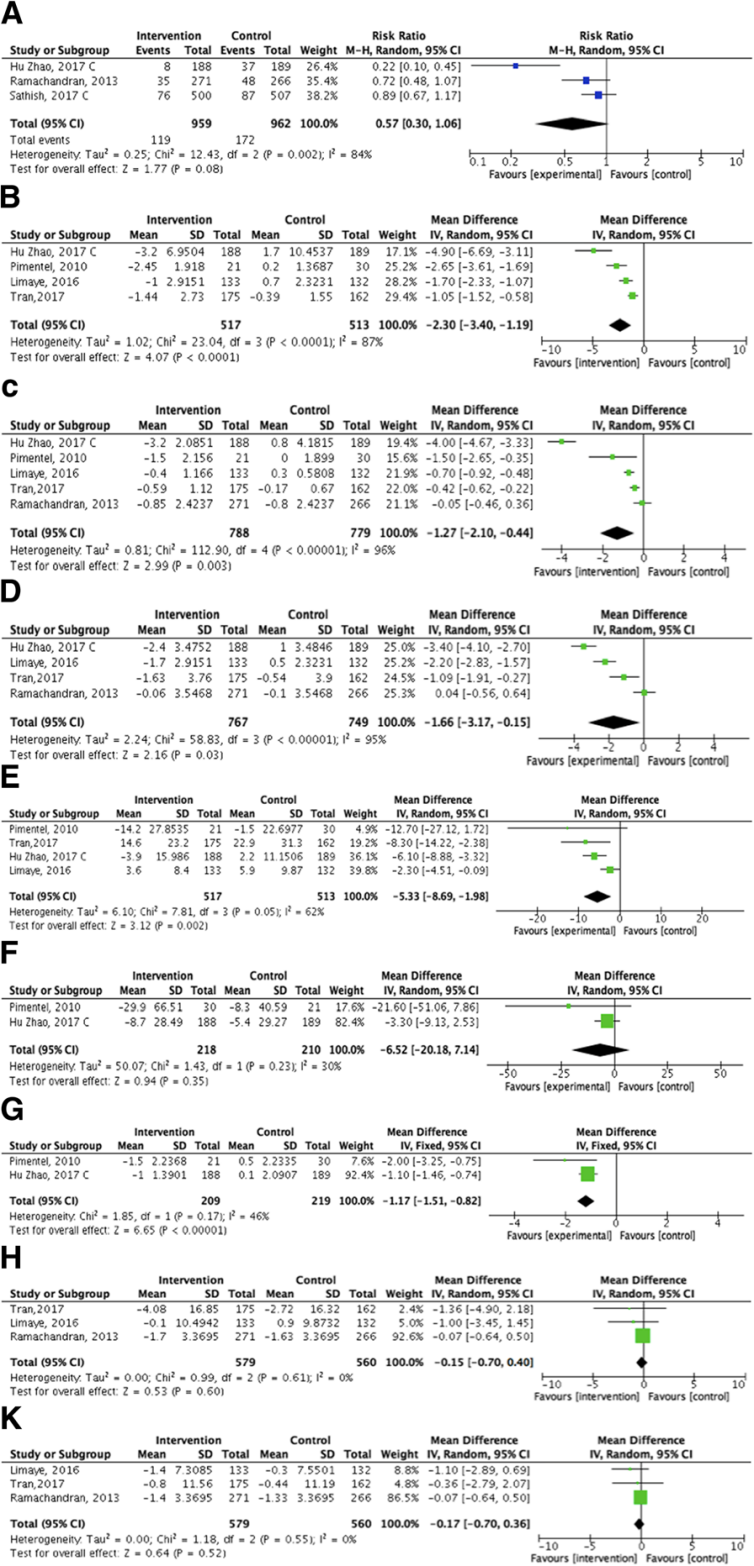
Meta-analyses of the intervention on the primary and secondary outcomes (**a**) cumulative incidence of type 2 diabetes; (**b**) Anthropometric indices; Weight change (kg); (**c**) Anthropometric indices; BMI change (kg/m2); (**d**) Anthropometric indices; Waist circumference change (cm); (**e**) Glycemic control change; Fasting blood glucose (mg/dl); (**f**) Glycemic control change; 2-h blood glucose (mg/dl); (**g**) Glycemic control change; HbA1C (%); (**h**) Blood pressure change; Systolic blood pressure; and (**k**) Blood pressure change; Diastolic blood pressure

Three trials reported the incidence rate of diabetes [67–69]; two of them were cluster randomized trial [68, 69]. We compared the results of the incidence rate of diabetes at 12 months follow-up for two trials [67, 69] and 24 months for one trial [68]. Based on the pooled effect, a total of 119 out of 959 participants developed T2DM in the intervention groups versus 172 out of 962 participants in the comparator groups. The calculated absolute effect is 77 fewer people per 1000 (95% CI from 173 fewer to 11 more) with the intervention. The RR (0.57 [0.30, 1.06]; *p* = 0.08) showed the risk of developing diabetes reduced by over 40% percent in favor of the community-based programs, but the difference was not statistically significant (Fig. 4 a).

#### Secondary outcomes

##### Anthropometric indices

Four studies reported weight (kg) at baseline and post-intervention [66, 69–71]. Twelve-month follow-up values were assessed in the quantitative analysis except for Tran et al., [71] where the maximum duration of follow-up was 6 months. There was a significant loss of more than 2 kg weight in favor of the community-based programs from baseline, (MD [95% CI]; − 2.30 [− 3.40, − 1.19], *p* = 0.00); Fig. 4 b. Five studies reported BMI (kg/m2) [66, 67, 69–71]. The pooled effect showed a significant reduction in BMI in favor of the interventions for 1567 participants (MD [95% CI]; − 1.27 [− 2.10, − 0.44]), *p* = 0.003; Fig. 4 c. Waist circumference (cm) was reported in four studies [66, 67, 69, 71]. The data of 12 months follow-up for two trials [66, 69] 2 years follow-up [67] and 6 months follow-up for the other trials [71] were entered for the meta-analysis. The pooled effect showed a significant reduction in favour of the interventions for 1516 participants (MD [95% CI]; − 1.66 [− 3.17, − 0.15], *p* = 0.03). See Fig. 4 d.

##### Glycemic control

Four trials reported fasting blood glucose (mg/dl) in the baseline and after the intervention [66, 69–71]. The pooled effect showed a statistically significant reduction in the values for 1030 participants (MD [95% CI]; − 5.33 [− 8.69, − 1.98]), *p* = 0.002); Fig. 4 e. Removing the trial at high risk of bias [70], showed the intervention still decreased the FBS (MD [95% CI]; − 4.94 [− 8.33, − 1.55], *p* = 0.004). Two trials reported 2-h blood glucose values (mg/dl) before and after 12 months of intervention [69, 70]. The result showed the intervention had no impact on the 2-h blood glucose values for 428 participants (MD [95% CI]; − 6.52 [− 20.18, 7.14], *p* = 0.35); Fig. 4 f. Two trials measured HbA1C (%) at baseline and 12 months [69, 70]. The pooled effect showed a significant difference in HbA1C measurements in favour of the intervention for 428 participants (MD [95% CI]; − 1.17 [− 1.51, − 0.82], *p* = 0.000); Fig. 4 g.

##### Blood pressure (mmHg)

Three studies (total 1139 participants) reported systolic and diastolic blood pressures (SBP and DBP) [66, 67, 71]. The pooled results indicated that the intervention had no effect on blood pressure. For both SBP and DBP change, the pooled effect showed no significant difference between two groups (MD [95% CI]; − 0.15 [− 0.70, 0.40], *p* = 0.6), Fig. 4 h, and (MD [95% CI]; − 0.17 [− 0.70, 0.36], *p* = 0.52), Fig. 4 k, respectively. No study was at high risk of bias or randomized cluster participants.

##### Sensitivity analysis and subgroup analysis

No study was at high risk of bias in the pooled effect of incidence of diabetes, waist circumference, SBP and DBP. Thus, we did not need to carry out the sensitivity analysis to exclude the effect of risk of bias for these outcomes. In the remaining outcomes, we carried out the sensitivity analysis by removing the trial at high risk of bias [70] from the pooled effect of 2-h blood glucose and HbA1C. The remaining trial [69] (cluster RCT) showed no significant difference of 2-h blood glucose (*p* = 0.27) between groups and a significant reduction in HbA1C (*p* = 0.000), in favour of the intervention group (*n* = 377). In weight and BMI pooled results also removing the only trial [70] at high risk of bias of allocation concealment did not alter the significant weight and BMI reduction in the intervention groups (MD [95% CI]; − 2.20 [− 3.52, − 0.87], *p* = 0.001, I^2^ = 89%) and (MD [95% CI]; − 1.23 [− 2.14, − 0.31], *p* = 0.009, I^2^ = 97%), respectively. There were insufficient trials to undertake subgroup analyses, though we were unable to carry out subgroup analysis to compare cluster randomized trials versus individual randomized trials.

##### Assessment of heterogeneity

Although the pooled results of most outcomes showed statistically significant changes in favour of the community-based interventions, there was evidence of considerable statistically significant between-trial heterogeneity in some pooled effects. I^2^ was above 80% in incidence of diabetes and anthropometric indices, and higher than 50% in FBS (see Fig. 3) that is considerable. To investigate the heterogeneity, we evaluated the effect of removing the high risk of bias trial [70] in anthropometric indices, removing the cluster-randomized trial [69], or removing both of them in the FBS results. We could not reduce the observed heterogeneity in any anthropometric indices, incidence of diabetes, or FBS pooled effects by eliminating the risk of bias, cluster effect, or both. Therefore, we considered the differences in duration and methods of the interventions as an explanation for the between-trials heterogeneity.

### Quality of evidence

**Fig. 5:**
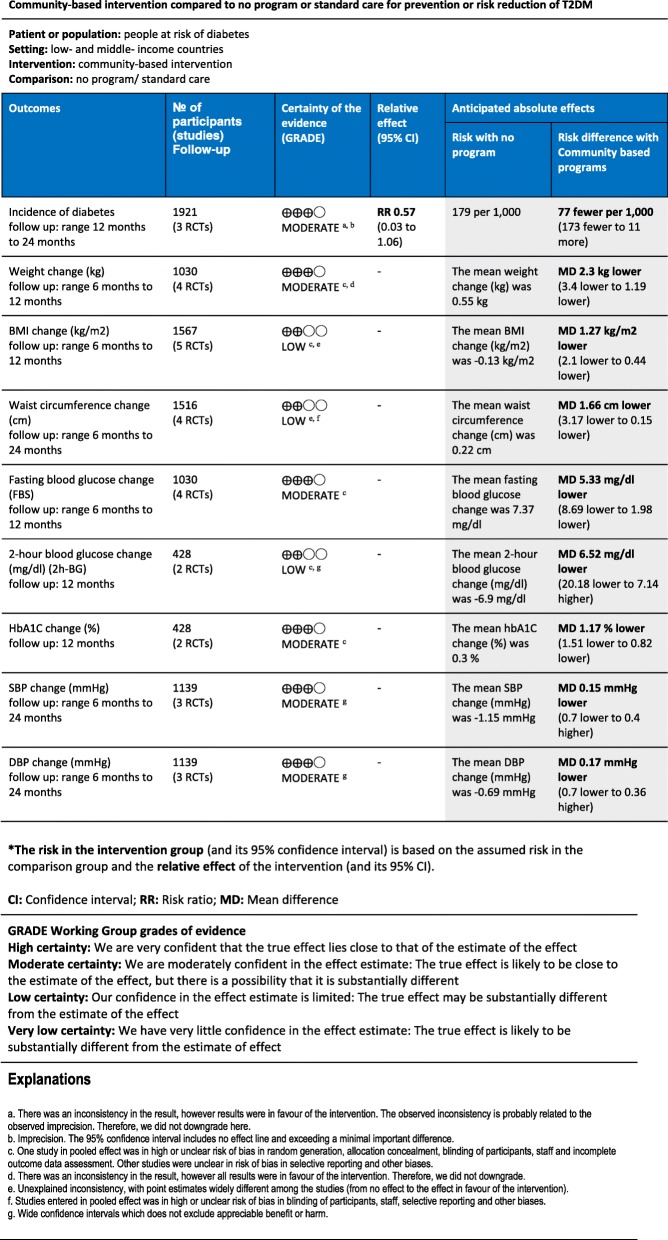
Summary of findings

We judged the quality of the evidence based on the GRADE approach and presented the evidence in Fig. 5. The directness of the evidence was judged to be satisfactory. However, we observed imprecision in the quality of evidence for several outcomes that resulted in one level downgrading in the quality of evidence for them. Also, there was evidence of considerable between-trial heterogeneity in the pooled results of some outcomes, which caused downgrading in case of unexplained heterogeneity. Some outcomes also were judged to have serious risk of bias based on our assessments in the previous section. For the incidence rate of diabetes, quality was downgraded by one level for imprecision but not an additional level for heterogeneity, since all results were in favor of the intervention and we had enough events to observe the effect of the intervention. The quality of evidence for the secondary outcomes was judged to be moderate- or low-quality regarding serious risk of bias or inconsistency or imprecision that resulted in some uncertainty in the evidence (See Fig. 5 footnotes).

## Discussion

This review summarized the evidence of six randomized controlled trials conducted in the last 10 years with 2574 participants analyzed to assess the effect of community-based interventions on the primary prevention of T2DM in low- and middle-income countries (LMICs). Overall, these results are optimistic that community-based interventions can modify several risk factors for T2DM, including anthropometric indices (weight, BMI, and waist circumference) and glycemic control (fasting blood glucose and HbA1C), and may be effective in risk reduction of T2DM in communities as well. Our findings indicated that the risk of developing T2DM (the incidence rate of diabetes) was lower in intervention groups, with a relative risk reduction of 0.57 (95% CI; 0.03 to 1.06). Therefore, 77 per 1000 fewer people developed diabetes after participating in these interventions (See Fig. 5). However, this moderate-quality evidence was not statistically significant, probably due to the heterogeneity that resulted in the wide confidence intervals.

In anthropometric indices, our analysis demonstrates that community-based programs probably have a beneficial effect on weight, BMI, and waist circumference. The pooled results of moderate-quality evidence showed a significant reduction of 2.3 kg weight (1.19 to 3.4 kg lower). In addition, our results revealed a reduction of 1.27 kg/m^2^ BMI (0.44 to 2.1 kg/m^2^ lower) and 1.66 cm in waist circumference. However, the quality of the evidence was low in these to outcomes due to inconsistency among the results of our studies and risk of bias in some of them. Two glycemic control outcomes also showed a significant improvement. HbA1C and FBS reduced significantly by 1.17% (1.51 to 0.82 lower) and 5.3 mg/dl (1.98 to 8.69 lower), respectively. The effect of community-based programs on blood pressure was limited in our review with wide thresholds that did not exclude appreciable benefit or harm.

The observed heterogeneity in the pooled effects of some outcomes is probably related to differences in methods and duration of the interventions. The interventions in our included studies used a variety of approaches ranging from physical activity and dietary interventions to lifestyle education through cell-phone text messages. The difference in the intensity of the interventions is expected to affect their effectiveness in participants’ lifestyle change. The duration of these interventions varied from 6 months to 2 years. Due to the low number of RCTs, we could not perform a subgroup analysis based on the duration of interventions to compare the effect of long-term interventions with short-term ones.

Although our review addresses the effect of community-based programs in populations at risk of diabetes only in LMICs, it highlighted several remarkable findings that confirm earlier reviews of lifestyle-related interventions on the prevention or risk reduction of T2DM in the world (HICs and LMICs). In 2012 Rawal L.B., et al., [29] assessed the effect of lifestyle-related interventions in developing countries on the incidence rate of diabetes, though their review did not carry out a meta-analysis and only reported the results of each trial. Similar to our findings of a descriptively lower relative risk reduction in intervention groups, they reported that lifestyle interventions can result in significant reductions of risk of the development of T2DM in people with impaired glucose tolerance or impaired fasting glucose. In China [10, 13], a 42% relative risk reduction of T2DM was reported with a diet and exercise intervention, comparable to our pooled effect T2DM risk reduction of 43%. Kerrison et al. [72] assessed nine trials and reported that lifestyle interventions reduced the incidence of diabetes more than standard treatment (in 8 of 9 studies reviewed) and increased weight reduction, but no meta-analysis was performed. In relation to anthropometric outcomes, Dunkley et al. (2014) [28] reviewed 22 experimental and observational studies of lifestyle interventions aimed at risk reduction or prevention of T2DM with the primary outcome of weight change. In contrast to our review, all studies were carried out in HICs (Europe and North America). Their pooled results showed a mean weight loss of 2.32 kg (95% CI [− 2.92, − 1.72]), very similar to our findings indicating a significant loss of 2.3 kg (95% CI [− 3.40, − 1.19]). Zhang [73] showed a 3.99% reduction in weight (95% CI [− 4.69, − 3.29]) in their review that is also similar to our results. In relation to glycemic control indicators, Qing-Hai Gong in 2015 reviewed nine trials (two from LMICs) and reported that lifestyle modification programs (physical or dietary interventions or both) were associated with significant improvements in 2-h blood glucose (SMD [95% CI]; − 0.56 [− 1.01 to − 0.10], *p* = 0.000) and FBS levels (SMD [95% CI]; − 0.27; [− 0.38 to − 0.15], *p* = 0.042) in patients with impaired glucose tolerance (IGT) [74]. Their result is in contrast with ours in relation to the significant decline of 2-h blood glucose. However, the FBS pooled result is comparable to our study findings. A review by Zhang [73] had similar findings showing lifestyle interventions reduced FBS and HbA1C significantly with FBS (MD [95% CI]; − 0.14 mmol/L [− 0.19, − 0.10]), and HbA1c (MD [95% CI]; − 0.06% [− 0.09, − 0.03]). One systematic review [75] including eight trials found that interventions combining physical activity and diet or behavioral modification in LMICs significantly reduced both the systolic blood pressure (SBP) (MD; 95%CI, − 6.1 mmHg; − 8.9 to − 3.3) and diastolic blood pressure (DBP) (MD; 95% CI -2.4 mmHg; − 3.7 to − 1.1). This result is different from our finding that interventions had no or limited effect on SBP and DBP. However, they found that the interventions were effective in lowering SBP and DBP only in the studies where participants received antihypertensive drugs. In our review, none of the three trials which reported the effect on blood pressure reported using antihypertensive drugs.

Overall, similar to our results, the previous systematic reviews’ findings suggest that behavioral, educational, or lifestyle modification interventions – such as community-based interventions – maybe effective to prevent or reduce the risk of T2DM in many countries, including LMICs.

### Limitations and potential biases in the review

We conducted our review based on the recommendations provided in the Cochrane Handbook for Systematic Review of Interventions [33] with a comprehensive literature search across major databases and two trial registries to identify published and unpublished trials. However, we did not conduct a broad search for gray literature and we limited our search results to English language literature. We planned to use funnel plots to evaluate potential publication bias if we included at least 10 trials, but due to the limited number of included RCTs (6 studies), we were not able to produce a funnel plot to assess publication bias that is the study limitation. Some of the included trials were relatively small and mostly described one-year follow-up, and some did not report all of our outcomes of interest. The limited number of included trials that reported each outcome made it impossible to carry out subgroup analysis.

The generalizability of our review is limited by the small number of included trial settings, age and condition of the participants, and method of intervention delivery. Half of the included trials were carried out in India and the other three in China, Brazil, and Vietnam. Thus, our review did not provide evidence across most LMICs. The recruited participants’ ages ranged from 30 to 76 years old. Therefore, our review result may not be applicable to younger adults, though T2DM is relatively rare among that age group. The content of interventions was reasonably similar, but the method of interventions varied from participation in a walking group and educational sessions, to cellphone text messages, to delivery of health behavioral modification. Since educational methods were different in the interventions, we cannot apply their results to all community-based programs.

## Conclusion

Regarding the evidence presently available, we can conclude that community-based interventions are probably effective in LMICs for the risk reduction of some modifiable risk factors of T2DM. These programs may have an impact on the incidence rate of diabetes and probably affect positively anthropometric indices and glycemic control in the at-risk population. However, due to the heterogeneity observed between trials, we suggest that more well-designed RCTs with longer follow-up duration are required to confirm whether community-based interventions lead to reduced T2DM events in the at-risk population of LMIC settings. The applicability of our findings may be limited, due to the few trials conducted in the handful number of low- and middle-income countries. Therefore, there is a need for rigorous randomized trials in many LMICs to fill this research gap and confirm the results. Further research is also needed to identify which type of community-based interventions with which modes of delivery can have the most effective impact on the prevention or risk reduction of T2DM.

## Additional files

Additional file 1: Search strategy. (PDF 228 kb)

Additional file 2: Characteristics of excluded studies. (PDF 39 kb)

## Abbreviations

BMI: Body mass index
CI: Confidence interval
CVD: Cardiovascular disease
DBP: diastolic blood pressures
FBS: fasting blood glucose
GRADE: Grading of Recommendations, Assessment, Development and Evaluation
HbA1C: Glycated hemoglobin A1
HICs: high income countries
ICC: intraclass correlation coefficient
IFG: Impaired Fasting Glucose
IGT: Impaired Glucose Tolerance
LMICs: low- and middle-income countries
MD: mean difference
PRISMA: Preferred Reporting Items for Systematic Reviews and Meta-Analyses
RCTs: randomized controlled trials
SBP: systolic blood pressures
SMD: standardized mean difference
T2DM: type 2 diabetes mellitus
