# Supplementary material for: The effect of community-based programs on diabetes prevention in low- and middle-income countries: a systematic review and meta-analysis
Source: Global Health. 2019 Feb 1;15:10. doi: 10.1186/s12992-019-0451-4 (PMC6359819; doi:10.1186/s12992-019-0451-4)
Supplement: Supplementary file 1 — Search strategy. (PDF 228 kb) [file 12992_2019_451_MOESM1_ESM.pdf]

Additional file 1: Search strategy

Database: Embase <1974 to 2018 March 06>
Search Strategy:
----
1 exp diabetes mellitus/ or diabet*.mp. (977380)
2 (diabet* adj5 risk).tw. (65678)
3 (diabet* adj5 prevent*).tw. (23120)
4 1 or 2 or 3 (977380)
5 exp health education/ (289049)
6 exp health behavior/ (353186)
7 exp counseling/ (143393)
8 primary prevention/ (35599)
9 exp preventive health service/ or preventive health service*.mp. (26491)
10 ((lifestyle or life-style or behavio?r*) adj3 (interven* or educat* or advice* or alter* or change* or inform*)).tw. (131435)
11 ((health* or wellness or weight or diet* or physical activit* or exercise) adj2 (promot* or program* or campaign* or advic* or educat* or awareness or therap*)).tw. (197593)
12 ((community* or population*) adj2 (program* or interven* or campaign* or educat*)).tw. (28416)
13 exp community care/ (113562)
14 5 or 6 or 7 or 8 or 9 or 10 or 11 or 12 or 13 (1058639)
15 developing country.sh,hw. or ((developing or less* developed or under developed or underdeveloped or middle income or low* income or underserved or under served or deprived or poor*) adj (countr* or nation? or population? or world)).ti,ab. (152203)
16 ((developing or less* developed or under developed or underdeveloped or middle income or low* income) adj (economy or economies)).ti,ab. (541)
17 exp Africa/ (286365)
18 exp asia, central/ or exp asia, northern/ or exp cambodia/ or exp indochina/ or exp timor-leste/ or exp indonesia/ or exp laos/ or exp malaysia/ or exp mekong valley/ or exp myanmar/ or exp philippines/ or exp thailand/ or exp vietnam/ or exp bangladesh/ or exp bhutan/ or exp india/ or exp afghanistan/ or exp iran/ or exp iraq/ or exp jordan/ or exp lebanon/ or exp syria/ or exp turkey/ or exp yemen/ or exp nepal/ or exp pakistan/ or exp sri lanka/ or exp china/ or exp "democratic people's republic of korea"/ or exp mongolia/ (876369)
19 exp cuba/ or exp dominica/ or exp dominican republic/ or exp haiti/ or exp jamaica/ or exp martinique/ or exp central america/ or exp mexico/ or exp argentina/ or exp bolivia/ or exp brazil/ or exp colombia/ or exp ecuador/ or exp french guiana/ or exp guyana/ or exp paraguay/ or exp peru/ or exp suriname/ or exp venezuela/ (214922)
20 exp albania/ or exp "bosnia and herzegovina"/ or exp bulgaria/ or exp kosovo/ or exp "macedonia (republic)"/ or exp moldova/ or exp montenegro/ or exp "republic of belarus"/ or exp romania/ or exp serbia/ or exp ukraine/ or exp transcaucasia/ or exp armenia/ or exp "georgia (republic)"/ (161806)
21 exp "federated states of micronesia"/ or exp fiji/ or exp marshall islands/ or exp papua new guinea/ or exp solomon islands/ or exp timor-leste/ or exp tonga/ or exp tuvalu/ or exp vanuatu/ (8619)
22 (cambodia*/ or Kampuchea/ or "north korea*"/ or myanmar/ or burma/ or burmese/ or fiji*/ or indonesia*/ or micronesia*/ or kiribati/ or laos/ or "marshall island*"/ or mongolia*/ or "Papua New Guinea"/ or Philippines/ or filipino*/ or samoa*/ or "Solomon Islands"/ or Timor-Leste/ or Melanesia*/ or tonga*/ or vanuatu/ or vietnam*/ or china/ or chinese/ or malaysia*/ or belau/ or pelew/ or Thailand/ or thai*/ or tuvalu/ or "ellice islands"/ or kyrgyzstan/ or kyrgyz*/ or kirghizia/ or kirghiz*/ or tajikistan/ or tadzhik/ or tadzhikistan/ or tajikistan/ or albania*/ or armenia*/ or georgia*/ or Jugoslavija*/ or Yugoslavia*/ or serbo-croat*/ or macedonia*/ or sloven*/ or kosovo/ or moldova*/ or ukrain*/ or Uzbekistan/ or Azerbaijan*/ or belarus/ or byelarus/ or belorussia/ or bosnia*/ or Bulgaria*/ or Kazakhstan/ or kazakh*/ or latvia*/ or montenegro/ or romania*/ or russia*/ or ussr/ or soviet/ or cccp/ or serbia*/ or turk*/ or haiti/ or belize/ or bolivia*/ or "el salvador"/ or guatemala*/ or guyana*/ or hondura*/ or nicaragua/ or paraguay/ or antigua/ or barbuda/ or Argentin*/ or brazil*/ or colombia*/ or "costa rica*"/ or cuba*/ or dominica*/ or ecuador*/ or grenad*/ or jamaica*/ or mexic*/

or panama*/ or peru*/ or "st lucia*"/ or "saint lucia*"/ or grenadines/ or surinam*/ or uruguay/ or venezuela*/ or djibouti/ or egypt*/ or iraq*/ or morocc*/ or Syria*/ or gaza*/ or yemen*/ or algeria*/ or iran*/ or jordan*/ or leban*/ or libya*/ or tunisia*/ or afghan*/ or bangladesh*/ or nepal*/ or bhutan*/ or india*/ or pakistan*/ or "sri lanka*"/ or maldiv*/ or benin/ or dahomey/ or "brukina faso"/ or "burkina fasso"/ or "upper volta"/ or burundi*/ or "Central African Republic"/ or Ubangi-Shari/ or african*/ or chad/ or comoros/ or comores/ or congo*/ or zaire/ or eritrea*/ or Ethiopia*/ or gambia*/ or Guinea*/ or Guinea-Bissau/ or "Portuguese Guinea"/ or kenya*/ or liberia*/ or Madagasca*/ or "Malagasy Republic"/ or malawi*/ or nyasaland/ or mali*/ or mauritania*/ or mozambi*/ or "portuguese east africa"/ or Niger/ or Rwanda*/ or Ruanda*/ or "sierra leone*"/ or somali*/ or tanzania*/ or togo*/ or uganda*/ or zimbabwe*/ or rhodesia*/ or cameroon*/ or "cape verde*"/ or "congo*or Cote d'Ivoire"/ or "Ivory Coast"/ or ghan*/ or "gold coast"/ or Lesotho/ or Basutoland/ or nigeria*/ or senegal*/ or sudan*/ or swazi*/ or zambia*/ or angola*/ or botswana*/ or Bechuanaland/ or Kalahari/ or gabon*/ or Mauriti*/ or "Agalega Islands"/ or namibia*/ or "south africa*"/) not (Aspergillus or Peptococcus or Schizothorax or Cruciferae or Gobius or Lasius or Agelastes or Melanosuchus or radish or Parastromateus or Orius or Apergillus or Parastromateus or Stomoxys or "New Guinea" or "Guinea Pig*" or "Guinea Fowl").mp. (325208)
23 15 or 16 or 17 or 18 or 19 or 20 or 21 or 22 (1608020)
24 random$.tw. (1274326)
25 factorial$.tw. (32151)
26 crossover$.tw. (65034)
27 cross over$.tw. (28878)
28 cross-over$.tw. (28878)
29 placebo$.tw. (269336)
30 (doubl$ adj blind$).tw. (186741)
31 (singl$ adj blind$).tw. (20688)
32 assign$.tw. (331239)
33 allocat$.tw. (124501)
34 volunteer$.tw. (229440)
35 crossover procedure/ (54524)
36 double blind procedure/ (146899)
37 randomized controlled trial/ (489746)
38 single blind procedure/ (30519)
39 24 or 25 or 26 or 27 or 28 or 29 or 30 or 31 or 32 or 33 or 34 or 35 or 36 or 37 or 38 (1970074)
40 4 and 14 and 23 and 39 (1590)
41 (exp animal/ or nonhuman/) not exp human/ (6211928)
42 40 not 41 (1584)
43 limit 42 to yr="2008 -Current" (1347)
44 limit 43 to english language (1287)

Database: OVID Medline Epub Ahead of Print, In-Process & Other Non-Indexed Citations, Ovid MEDLINE(R) Daily and Ovid MEDLINE(R) 1946 to 2018 March 06
Search Strategy:
----
1 exp diabetes mellitus/ or exp diabetes mellitus, experimental/ or exp diabetes mellitus, type 2/ or exp hyperglycemia/ or exp metabolic syndrome/ (412241)
2 (diabet* adj5 risk).mp. [mp=title, abstract, original title, name of substance word, subject heading word, keyword heading word, protocol supplementary concept word, rare disease supplementary concept word, unique identifier, synonyms] (43181)
3 (diabet* adj5 prevent*).mp. [mp=title, abstract, original title, name of substance word, subject heading word, keyword heading word, protocol supplementary concept word, rare disease supplementary concept word, unique identifier, synonyms] (16640)
4 1 or 2 or 3 (429147)

5 exp "early intervention (education)"/ or exp health education/ or exp primary prevention/ or exp secondary prevention/ (372356)
6 (early intervention or health education or primary prevention or secondary prevention).mp. (150491)
7 ((lifestyle or life-style or behavio?r*) adj3 (interven* or educat* or advice* or alter* or change* or inform*)).tw. (101988)
8 ((health* or wellness or weight or diet* or physical activit* or exercise) adj2 (promot* or program* or campaign* or advic* or educat* or awareness or therap*)).tw. (160896)
9 heath behavior.mp. or exp Health Behavior/ (275549)
10 ((community* or population*) adj2 (program* or interven* or campaign* or educat*)).mp. [mp=title, abstract, original title, name of substance word, subject heading word, keyword heading word, protocol supplementary concept word, rare disease supplementary concept word, unique identifier, synonyms] (23753)
11 5 or 6 or 7 or 8 or 9 or 10 (835848)
12 exp asia, central/ or exp asia, northern/ or exp cambodia/ or exp indochina/ or exp timor-leste/ or exp indonesia/ or exp laos/ or exp malaysia/ or exp mekong valley/ or exp myanmar/ or exp philippines/ or exp thailand/ or exp vietnam/ or exp bangladesh/ or exp bhutan/ or exp india/ or exp afghanistan/ or exp iran/ or exp iraq/ or exp jordan/ or exp lebanon/ or exp syria/ or exp turkey/ or exp yemen/ or exp nepal/ or exp pakistan/ or exp sri lanka/ or exp china/ or exp "democratic people's republic of korea"/ or exp mongolia/ (443565)
13 exp AFRICA/ (230815)
14 exp cuba/ or exp dominica/ or exp dominican republic/ or exp haiti/ or exp jamaica/ or exp martinique/ or exp central america/ or exp mexico/ or exp argentina/ or exp bolivia/ or exp brazil/ or exp colombia/ or exp ecuador/ or exp french guiana/ or exp guyana/ or exp paraguay/ or exp peru/ or exp suriname/ or exp venezuela/ (172179)
15 exp albania/ or exp "bosnia and herzegovina"/ or exp bulgaria/ or exp kosovo/ or exp "macedonia (republic)"/ or exp moldova/ or exp montenegro/ or exp "republic of belarus"/ or exp romania/ or exp serbia/ or exp ukraine/ or exp transcaucasia/ or exp armenia/ or exp "georgia (republic)"/ (41954)
16 exp Pacific Islands/ (54610)
17 (cambodia*/ or Kampuchea/ or "north korea*"/ or myanmar/ or burma/ or burmese/ or fiji*/ or indonesia*/ or micronesia*/ or kiribati/ or laos/ or "marshall island*"/ or mongolia*/ or "Papua New Guinea"/ or Philippines/ or filipino*/ or samoa*/ or "Solomon Islands"/ or Timor-Leste/ or Melanesia*/ or tonga*/ or vanuatu/ or vietnam*/ or china/ or chinese/ or malaysia*/ or belau/ or pelew/ or Thailand/ or thai*/ or tuvalu/ or "ellice islands"/ or kyrgyzstan/ or kyrgyz*/ or kirghizia/ or kirghiz*/ or tajikistan/ or tadzhik/ or tadzhikistan/ or tajikistan/ or albania*/ or armenia*/ or georgia*/ or Jugoslavija*/ or Yugoslavia*/ or serbo-croat*/ or macedonia*/ or sloven*/ or kosovo/ or moldova*/ or ukrain*/ or Uzbekistan/ or Azerbaijan*/ or belarus/ or byelarus/ or belorussia/ or bosnia*/ or Bulgaria*/ or Kazakhstan/ or kazakh*/ or latvia*/ or montenegro/ or romania*/ or russia*/ or ussr/ or soviet/ or cccp/ or serbia*/ or turk*/ or haiti/ or belize/ or bolivia*/ or "el salvador"/ or guatemala*/ or guyana*/ or hondura*/ or nicaragua/ or paraguay/ or antigua/ or barbuda/ or Argentin*/ or brazil*/ or colombia*/ or "costa rica*"/ or cuba*/ or dominica*/ or ecuador*/ or grenad*/ or jamaica*/ or mexic*/ or panama*/ or peru*/ or "st lucia*"/ or "saint lucia*"/ or grenadines/ or surinam*/ or uruguay/ or venezuela*/ or djibouti/ or egypt*/ or iraq*/ or morocc*/ or Syria*/ or gaza*/ or yemen*/ or algeria*/ or iran*/ or jordan*/ or leban*/ or libya*/ or tunisia*/ or afghan*/ or bangladesh*/ or nepal*/ or bhutan*/ or india*/ or pakistan*/ or "sri lanka*"/ or maldiv*/ or benin/ or dahomey/ or "brukina faso"/ or "burkina fasso"/ or "upper volta"/ or burundi*/ or "Central African Republic"/ or Ubangi-Shari/ or african*/ or chad/ or comoros/ or comores/ or congo*/ or zaire/ or eritrea*/ or Ethiopia*/ or gambia*/ or Guinea*/ or Guinea-Bissau/ or "Portuguese Guinea"/ or kenya*/ or liberia*/ or Madagasca*/ or "Malagasy Republic"/ or malawi*/ or nyasaland/ or mali*/ or mauritania*/ or mozambi*/ or "portuguese east africa"/ or Niger/ or Rwanda*/ or Ruanda*/ or "sierra leone*"/ or somali*/ or tanzania*/ or togo*/ or uganda*/ or zimbabwe*/ or rhodesia*/ or cameroon*/ or "cape verde*"/ or "congo*or Cote d'Ivoire"/ or "Ivory Coast"/ or ghan*/ or "gold coast"/ or Lesotho/ or Basutoland/ or nigeria*/ or senegal*/ or sudan*/ or swazi*/ or zambia*/ or angola*/ or botswana*/ or Bechuanaland/ or Kalahari/ or gabon*/ or Mauriti*/ or "Agalega Islands"/ or namibia*/ or "south africa*"/) not (Aspergillus or Peptococcus or Schizothorax or Cruciferae or Gobius or Lasius or Agelastes or Melanosuchus or radish or Parastromateus or Orius or Apergillus or Parastromateus or Stomoxys or "New Guinea" or "Guinea Pig*" or "Guinea Fowl").mp. (300075)
18 "Developing Countries".sh,kf. or ((developing or less* developed or under developed or underdeveloped or middle income or low* income or underserved or under served or deprived or poor*) adj (countr* or nation? or

population?)).ti,ab. or ((developing or less* developed or under developed or underdeveloped or middle income or low* income) adj (economy or economies)).ti,ab. or (low* adj (gdp or gnp or gross domestic or gross national)).ti,ab. or (low adj3 middle adj3 countr*).ti,ab. or (lmic or lmics or "lami countr*").ti,ab. (132854)

19 12 or 13 or 14 or 15 or 16 or 17 or 18 (1080928)
20 4 and 11 and 19 (3008)
21 exp animals/ not humans.sh. (4430952)
22 20 not 21 (3006)
23 randomized controlled trial.pt. (454849)
24 controlled clinical trial.pt. (92204)
25 randomized.ab. (404382)
26 placebo.ab. (186843)
27 drug therapy.fs. (1997167)
28 randomly.ab. (285994)
29 trial.ab. (419979)
30 groups.ab. (1769051)
31 23 or 24 or 25 or 26 or 27 or 28 or 29 or 30 (4151259)
32 22 and 31 (1206)
33 limit 32 to yr="2008 -Current" (932)
34 limit 33 to english language (889)

Database: CENTRAL <Cochrane Library> 2018 March 06
Search Strategy:

ID	Search
#1	diabetes or diabet*
#2	MeSH descriptor: [Diabetes Mellitus] explode all trees
#3	diabet* near/5 risk
#4	diabet* near/5 (prevention or prevent*)
#5	#1 or #2 or #3 or #4
#6	MeSH descriptor: [Early Intervention (Education)] explode all trees
#7	MeSH descriptor: [Health Education] explode all trees
#8	MeSH descriptor: [Primary Prevention] explode all trees
#9	MeSH descriptor: [Secondary Prevention] explode all trees
#10	health promotion or health behavio?r or health education or Counseling
#11	((lifestyle or life-style or behavio?r*) near/3 (interven* or educat* or advice* or alter* or change* or inform*))
#12	((health* or wellness or weight or diet* or physical activit* or exercise) near/2 (promot* or program* or campaign* or advic* or educat* or awareness or therap*))
#13	(primary near/3 prevent*)
#14	((community* or population) near/2 (program* or interven*))
#15	#6 or #7 or #8 or #9 or #10 or #11 or #12 or #13 or #14
#16	Developing Countries
#17	((developing or "less* developed" or "under developed" or underdeveloped or "middle income" or "low* income" or underserved or "under served" or deprived or poor*) near (countr* or nation? or population? or world))
#18	((developing or "less* developed" or "under developed" or underdeveloped or "middle income" or "low* income") near (economy or economies))
#19	(low* near (gdp or gnp or "gross domestic" or "gross national"))
#20	(low near/3 middle near/3 countr*)
#21	(lmic or lmics or third world or lami countr*)
#22	#16 or #17 or #18 or #19 or #20 or #21
#23	#5 and #15 and #22 Publication Year from 2008 to 2018 (#1384)
