# Supplementary material for: The effect of community-based programs on diabetes prevention in low- and middle-income countries: a systematic review and meta-analysis
Source: Global Health. 2019 Feb 1;15:10. doi: 10.1186/s12992-019-0451-4 (PMC6359819; doi:10.1186/s12992-019-0451-4)
Supplement: Supplementary file 2 — Characteristics of excluded studies. (PDF 39 kb) [file 12992_2019_451_MOESM2_ESM.pdf]

Additional file 2: Characteristics of excluded studies

No	Study	Reason for exclusion
1	Luo Y, 2017 [42]	Wrong intervention; combined pharmaceutical and lifestyle intervention
2	D-CLIP (Weber M), 2012 [43]	Wrong intervention; More than 50% of participants in the intervention arm had been administrated Metformin
3	Chen MF, 2017 [44]	Improper intervention; Not community-based; Convenience sampling from a hospital in Taiwan
4	Lin A, 2014 [45]	Wrong intervention; combined pharmaceutical and lifestyle; intervention arm had been administrated diabetic medication
5	DIABRISK (Wijesuriya M), 2011 [46]	Improper intervention; same intervention applied to the intervention and control in the intensive vs. less-intensive way; entered population age 5 to 40 years old
6	Liu H, 2016 [47]	Wrong population; entered pregnant women with GDM
7	DIABRISK (Guess N), 2016 [48]	Improper intervention; same intervention applied to the intervention and control in the intensive vs. less-intensive way; entered population age 5 to 40 years old
8	Nanditha A, 2016 [49]	Pooled data of two studies: one study was not eligible (not community-based intervention that performed in 2006 and was not among our search result from 2008). the other study is one of our included studies.
9	Aira T, 2013 [50]	Improper intervention; wrong control program; Not community-based
10	Cheng S, 2017 [49] [51]	Improper population; Not community based study aimed to improve chronic liver disease among IGT patients with non-alcoholic fatty liver disease
11	De Azevedo MBG, 2017 [52]	Improper intervention; Not community-based
12	Dunkley AJ, 2011 [53]	Wrong outcome; only baseline data is presented
13	Xu DF, 2013 [54]	Improper intervention; Not community-based
14	Whittemore R, 2014 [55]	Wrong outcome; did not report our study outcomes
15	Toobert DJ 2011 [56]	Wrong setting; Latinas living in USA
16	Stepenka V, 2010 [57]	No outcome; only an abstract is available that did not present our study results.
17	Siqueira-Catania A, 2013 [58]	Improper intervention; Not community-based
18	Da Qing (Li G) 2014 [59]	Improper intervention; Not community-based
19	Da Qing (Li G) 2008 [60]	Improper intervention; Not community-based
20	Da Qing (Shen X), 2014 [61]	Improper intervention; Not community-based
21	Florez HJ, 2012 [62]	Wrong outcome; an abstract that did not present our study results
22	Jahangiry L, 2015 [63]	Improper intervention; Not community-based
23	Shek NWM, 2013 [64]	Improper intervention; Not community-based
24	Essien O, 2017 [65]	Improper population; entered T2DM patients
25	Lu Y-H, 2011 [66]	Wrong intervention; combined pharmaceutical and lifestyle intervention
26	Pichayapinyo P, 2015 [67]	Improper intervention; Not community-based
27	DIABRISK (Wijesuriya M) 2017 [68]	Improper intervention; same intervention applied in the intensive or less-intensive way; entered children and adolescents
28	Perichart-Perera O, 2014 [69]	Improper intervention; Not community-based
29	Wong KH, 2013 [70]	Wrong study setting; Hong Kong is classified HICs
30	Oba N, 2011 [71]	Inappropriate study design; No comparison group
